# Surfactant Effects on Microemulsion-Based Nanoparticle Synthesis

**DOI:** 10.3390/ma4010055

**Published:** 2010-12-29

**Authors:** Concha Tojo, Miguel de Dios, Fernando Barroso

**Affiliations:** Physical Chemistry Department, Faculty of Chemistry, University of Vigo, E-36310 Vigo, Spain; E-Mails: mdedios@uvigo.es (M.D.D.); ferbarroso@uvigo.es (F.B.)

**Keywords:** nanoparticles, microemulsions, simulation

## Abstract

The effect of the surfactant on the size, polydispersity, type of size distribution and structure of nanoparticles synthesized in microemulsions has been studied by computer simulation. The model simulates the surfactant by means of two parameters: the intermicellar exchange parameter, *k_ex_*, related to dimer life time, and film flexibility parameter, *f*, related to interdroplet channel size. One can conclude that an increase in surfactant flexibility leads to bigger and polydisperse nanoparticle sizes. In addition, at high concentrations, the same reaction gives rise to a unimodal distribution using a flexible surfactant, and a bimodal distribution using a rigid one. In relation to bimetallic nanoparticles, if the nanoparticle is composed of two metals with a moderate difference in reduction potentials, increasing the surfactant flexibility modifies the nanoparticle structure, giving rise to a transition from a nanoalloy (using a rigid film) to a core-shell structure (using a flexible one).

## 1. Introduction

Producing nanoparticles has received impressive attention in recent years because of their several non conventional physical properties (catalytic, optic, electric and magnetic) [1,2,3,4,5,6]. Although progress in this field has been extremely important, much has yet to be done in order to understand the properties of nanosized particles, and also to obtain better control of the nanostructure of these materials. Important features are the high surface energy and chemical reactivity of nanoparticles. Therefore, a variety of techniques have been used to prepare finely dispersed nanoparticles.

This paper focuses on nanoparticle synthesis via the reverse microemulsion method. This method has been employed for the preparation of nanoparticles from a diverse variety of materials, including metals [7,8,9,10], silica [11] and other oxides [9,12], polymers [13,14], semiconductors [15], superconductors [16] and particles with a core-shell structure [17,18,19,20]. Such dynamic colloidal templates are known to produce particles of smaller size than those obtained via normal precipitation in aqueous systems. Microemulsions consist of nanometer-size water droplets which are dispersed in a continuous oil medium and stabilized by surfactant molecules accumulated in the oil-water interface. The main function of the droplet nanoreactor is to provide a compartmentalized medium in order to prevent phase separation of the particles. In fact, water-in-oil microemulsions have successfully been used to produce a variety of nanoparticle shapes and sizes [7,14,21]. Nevertheless, it is not known how such templates control the size and shape of the resulting materials, and therefore, this issue still requires further examination. Although microemulsion templating is known to produce particles of desirable properties, it appears that the crucial size-controlling parameters of nanoparticles is not only the template itself, and there must be other important factors. One of the key factors is the nature of adsorbed surfactant film [22,23,24].

This work is focused on the study of how the microemulsion composition affects the size, size distribution, monodispersity and structure of final nanoparticles.

## 2. Simulation Procedure

The computer simulation was run to simulate the kinetic course of the reaction.

### 2.1. Microemulsion Description

The microemulsion is represented as a set of droplets randomly located on a three dimensional lattice, which can move and collide with each other. Each simulation begins with a random distribution of the two or three sets of microemulsion droplets, depending on the kind of nanoparticle: if the simulated nanoparticle is a simple one, the one-pot method is simulated by mixing equal volumes of two microemulsions, one containing the reactant *A*, and other containing the reactant *B*, with the reaction *A + B → P*. To simulate the synthesis of a bimetallic nanoparticle, three microemulsions are mixed, one containing the metal salt *A*, the second containing the metal salt *B*, and the third containing the reducing agent *R*. In this paper, we present results considering a ϕ = 10% portion of the space occupied by droplets.

### 2.2. Initial Reactant Distribution

The reactant species were distributed throughout the droplets using a Poisson distribution. In this study, we present results using 〈*c*〉 units of each kind of reactant inside the droplets, *i.e.*, [A] = [B] = 〈*c*〉. In the case of bimetallic nanoparticles, the reducing agent concentration 〈*c_R_*〉 was always double that of the metal precursors’ concentrations.

### 2.3. Motion and Collision

In the algorithm used to simulate the simple nanoparticle obtention [25,26], droplets were allowed to perform random walks to nearest neighbor sites, by choosing at random the direction of the motion at each step. The length of each step was constant and equal to one length lattice unit. This random walk was subject to the exclusion principle, and cyclic boundary conditions were enforced at the ends of the lattice. Droplets collided when they occupied contiguous lattice sites. In order to save computation time, the model was improved by simulating the movement and collisions as follows: Two micelles chosen randomly are allowed to collide (due to Brownian motion), fuse and redisperse. Upon collision, they can establish a water channel forming a transient dimer (fusion), exchanging their contents (reactants, products and/or growing particles). Both ways of simulating the motion and collision lead to the same results [27]. The second method was used to simulate bimetallic nanoparticle synthesis because it is less time consuming.

### 2.4. Time Unit Base

Our time unit base is one Monte Carlo step (mcs), which was initially defined as the time it takes for all droplets to move to the nearest position in the lattice. By using the second description of the movement, 1 mcs is defined as the time needed for an *n* number of collisions to take place. The number of collision by unit time is directly related to the volume fraction occupied by droplets.

### 2.5. Intermicellar Exchange Criteria of Reactants

Regardless of the presence or absence of product, if both colliding droplets carried the same reactant, this reactant was redistributed in accordance with a crude concentration gradient principle: the reactant (metal salt or reducing agent) is transferred from the droplet with more reactants to the droplet with less reactants. The parameter *k*_ex,A_ determines how many units of reactant *A* could be transferred during a collision. If the more concentrated droplet carried a quantity of molecules greater than *k*_ex,A_, a maximum number *k*_ex,A_ units of reactant *A* could be interchanged to the droplet containing less reactants. After collision, the concentration inside the colliding droplets will be the same. The algorithm allow us to distinguish a different value for *k*_ex,A_, *k*_ex,B_ and *k*_ex,R_ for each kind of reactant (*A* and *B* to simulate simple particles, and *A*^+^, *B*^+^, and *R* to simulate bimetallic ones). In this way, the material nature, its size and electric charge, can be taken into account. In this paper, we present results using *k*_ex,A_ *=* *k*_ex,B_ *=* *k*_ex,R_ *=* *k*_ex_.

A different approach to describe the dynamics of a concentration distribution inside a droplet has been proposed by Curl [28], and successfully improved by Niemann *et al*. [29,30,31]. In this different approach, a multidimensional population balance equation model was derived [29], as well as a bivariate droplet exchange kernel for the distribution of reactants [31].

### 2.6. Chemical Reduction Rates

When two droplets containing different reactants collide and mass transfer takes place, both kinds of reactants can be located inside the same micelle, and chemical reaction is possible. In order to include different reactions rates, only a percentage of v of reactants inside the colliding droplets gives rise to products. The fastest reaction corresponds to v *=* 1 (100% reactants transform in products), *i.e.*, an instantaneous reaction is considered. By decreasing the value of v, the chemical reaction rate effect on the synthesis can be studied. The co-existence of reactant molecules inside the same micelle is allowed (reactants which did not react). They remain in the water-pool, and will be transferred or will react in a subsequent collision.

When a bimetallic nanoparticle synthesis is simulated, two different reaction rate parameters have to be considered because of there are two different metal salts in the reaction media, for which reduction can occur at different rates. The reduction of the metal salt *A*^+^ to obtain metal *A* (*A*^+^ + *R* → *A*) can be called v_A_, where v_A_ is the percentage of metal precursor *A*^+^ inside the colliding droplets which gives rise to metal atoms *A*. Likewise, the reduction of the metal salt *B*^+^ to obtain metal *B* (*B*^+^ + *R* → *B*) is determined by v_B_. In this study, we monitored results using reduction rate ratios from v_A_/v_B_ = 1 to v_A_/v_B_ = 100. In all cases, the *A* metal chemical reduction rate is assumed to be instantaneous (*i.e.*, 100% of reactants inside the same droplet give rise to products), so *A* metal can be called the quicker metal. The other chemical reduction is slowed down (10% or 1% *B*^+^ metal salt reduces to *B* metal atoms), so *B* is called the slower metal. The algorithm decides randomly which reaction occurs first when both reactions are possible in the same droplet.

### 2.7. Critical Nucleation Number and Intermicellar Exchange of Products

Nucleation is the process by which atoms (or ions), which are free in solution, come together to produce a thermodynamically stable cluster. According to La Mer’s homogeneous nucleation and growth model [32], there will be a critical nucleus size which is characteristic of the specific material considered. The cluster must exceed a critical size determined by the competition between the aggregate curvature (Laplace pressure) and the free energy favoring the growth of the new phase. Once the critical size is exceeded, the cluster becomes a supercritical nucleus capable of further growth. If the nucleus is smaller than the critical size, spontaneous dissolution can occur. This fact is simulated by including a parameter n* (the critical nucleus size), which is introduced in the simulation as follows: if the number of product molecules inside the same droplet is smaller than n*, they are considered free inside the droplet, so they can be interchanged during a subsequent collision; this interchange is governed by the *k*_ex,_*_P_* parameter defined below. On the contrary, when the number of products inside the same droplet is equal to or greater than n*, they come together, forming a stable nucleus. This nucleus can only be transferred as a whole during a posterior collision, and this exchange is governed by the *f* parameter (see below).

In the case of bimetallic nanoparticles, both metals can need a different minimum number of atoms to form a stable nucleus, capable of further growth, so the algorithm distinguishes two different critical nucleation number (n_A_* and n_B_*). In this paper we present results using n_A_* = n_B_* = n* = 1.

### 2.8. Intermicellar Exchange Criteria of Free Atoms/Molecules of Products

As has been noted before, when the number of products inside the same water pools does not reach the critical nucleus size value, they remain free (non-aggregated) and can be interchanged during a later collision. The product exchange parameter (*k*_ex,_*_P_*) governs this exchange, by determining how many atoms of product could be transferred during a collision. This quantity is related to the rate of product intermicellar exchange. In the case of bimetallic nanoparticles, the characteristics of each metal can allow passing the interdroplet channel easier or more difficult, so the interdroplet exchange parameter could be different. In this study, we only present results using the same value for all free units (reactants and products) *k_ex,P_* = *k_e__x_*.

Whenever a metal atom is interchanged to a droplet carrying a nucleus, this unit is added to the nucleus, giving rise to a growing particle (aggregate).

### 2.9. Autocatalysis

As the reaction takes place, more droplets could contain reactants and nuclei simultaneously. The interchange of reactants between two colliding droplets in the presence of a growing nucleus allows us to simulate an autocatalytic reaction as follows: when one of the droplets is carrying an aggregate, the reaction always proceeds on the aggregate and the reaction rate will be double. When both droplets are carrying aggregates, autocatalysis takes place on the bigger one, because larger particles have a larger surface, which increases the probability of catalyzing the reaction. We have described the case of reaction in the absence of autocatalysis previously [33]. This work is concerned with autocatalytic reactions.

### 2.10. Intermicellar Exchange Criteria of Growing Particles

As the reaction advances, the exchange of growing particles (aggregates of metals) becomes more important. At this stage, collisions between two droplets—both containing a growing nucleus—are the most probable. The interchange of reactants, free metal atoms and growing particles during the same collision is allowed. To explain the interchange criteria in this situation it is important to point out two aspects:

#### 2.10.1. Surfactant Film Flexibility

The shape and size of nanodroplets are governed by the free energy of curvature and are determined by the elastic constant and the curvature of the surfactant film [34]. The elasticity of the film, which determines the material interdroplet exchange, depends not only on the surfactant but also on the presence of additives and the length of the oil phase [24,35,36,37]. It is well-known that the material interdroplet exchange process includes the opening of the interfacial layer. To introduce this phenomenon in our simulation, we can relate the flexibility of the surfactant film around the droplets and the ease with which channels communicating colliding droplets can form. Surfactant film flexibility, therefore, also places a limit on the size of the particles traversing the droplet-droplet channels. The influence of surfactant film flexibility is taken into account by varying a flexibility parameter (*f*) specifying a maximum particle size for transfer between droplets: particles composed of more than *f* units are not allowed to pass from one droplet to another. In this way, a highly flexible film will allow the interchange of larger particles than a rigid film.

#### 2.10.2. Ripening

Many studies have been reported for different growing, aging, or ripening mechanisms in bulk [38,39]. A particular coarsening process that has to be considered in microemulsions is Ostwald ripening. The particle size changes by solubilization and condensation of material. Ostwald ripening assumes that the largest particles will grow by condensation of material, coming from the smallest particles that solubilize more readily than larger ones. The possibility of ripening has been introduced in the simulation as follows: if a droplet containing *i* units of product (*P_i_*, where *P* is one of the two metals) collides with another droplet with a higher number of *P* units (*P_j_*), the smaller aggregate can be interchanged during a collision from the initial droplet to the droplet carrying the larger one (*P_i_* + *P_j_ → P_i+j_*). This mass transfer will only be allowed if the channel size *f* is greater or equal to *P_i+j_*. This criterion is only dependent on the aggregate size, *i.e.*, it does not take into account the composition (*A* or *B* metals).

### 2.11. Droplet Size

The growth of nanoparticle via coagulation may be limited by the size of the drops because a substantial amount of energy would be required to increase the drop size, as the surfactant film covering the drop has a finite bending modulus. When the microemulsion technique was introduced, it was thought that this would allow particle size to be controlled just by controlling the droplet size. However, several different types of behavior have been observed when the droplet size increases: increasing of particle size [40,41,42], bimodal distributions [43,44], and essentially no control by droplet size [44,45,46]. Although water droplets are not real templates, the combination of the droplet size with the parameters discussed above can be used to obtain very precise control of the final particle size. The simulation allows the modification of the droplet size with the *q* parameter, which restricts the maximum number of products that can be carried by a droplet. This influence was studied previously [47], so in this work droplets do not restrict nanoparticle growth. The total concentration of metal ions under the present experiment is so small that the influence of droplet size has no great influence on the nanoparticle formation.

## 3. Model

It is a well-known experimental result that the intermicellar dynamics is affected by changing the microemulsion composition (surfactant, length of the oil phase, cosurfactant) [1,24,35,36,37,48,49]. A basic property of a surfactant film, called ‘flexibility’, is its ability to depart from the optimal curvature. Surfactants can be flexible or rigid, depending on the strength of the interactions at both sides of the interface. In the formulation of microemulsions where the free energy cost for creating the interfacial area has to be compensated by a large entropic term, a flexible surfactant allowing curvature fluctuations is required [50,51]. Surfactants which strongly adsorb at interfaces and efficiently lower the interfacial tension, have a very hydrophobic part (long alkyl chain) and very hydrophilic headgroup [52]; they are rigid surfactants.

For a better understanding of the surfactant’s role on the nanoparticle synthesis in microemulsions, we will focus our attention on two factors that depend on surfactant: the dimer stability, which depends on the intermicellar attractive potential, and the size of the channel communicating colliding droplets, which depends on the surfactant rigidity. Both factors have a great influence on mass transfer, and consequently, on the exchange rate constant [53].

Firstly, we will discuss how the dimer stability is included in the simulation. The largest the dimer stability, the longer two water pools stay together, and the more material can be transferred during an effective collision. The reactant exchange parameter, *k_ex_*, determines how many units of reactant and/or non-aggregated products could be interchanged during a collision. A high value of *k_ex_* would also imply that the droplets have a high tendency to stay together, *i.e.*, the stickiness parameter will be great. Therefore, the reactant exchange parameter *k_ex_* increases as the dimer stability increases, and consequently, the rate of intermicellar exchange.

The second point of interest is the size of the channel which communicates colliding droplets. It is well-known that the intermicellar exchange process includes the opening of the interfacial layer, governed by surfactant film flexibility. A highly flexible film will allow the interchange of larger aggregates of particles than a rigid film. We can include in this picture our film flexibility parameter *f*, which limits the size of the particles traversing droplet-droplet channels. As the size of the channel that communicates colliding droplets is proportional to the surfactant film flexibility, the fact that the formation of larger particles is favored by ripening at high *f* values [33] implies that the rate will be higher as *f* increases. Therefore, the effective rate constant for droplet communication will increase with *f.* In the model, the *f* parameter is related to channel size [54]: a value *f* = 30 would imply a “permeation channel” (the minimum section inside the fused dimer) of approximately 6–10 Å, assuming that 30 metal atoms are aggregated in a spherical shape. For *f* = 5, one can obtain a permeation channel of about 1–2 Å.

On the other hand, the interdroplet material exchange includes different species: reactants, products and aggregates of products. These species can be transferred from one droplet to another if the droplets stay enough time together and if the size of the channel communicating colliding droplets is large enough. However, the interchange depends on the nature of these species. Since a reactant molecule or free product molecule is smaller than an aggregate, it could be assumed that the main factor which determines the interdroplet transfer of free molecules (reactants and/or non-aggregated products) would be the dimer stability, and the channel size would not be so important in this case. On the contrary, the size channel becomes important when the interchanged material is a particle constituted by aggregation of some units of products, which have to be transferred as a whole.

From the most simple picture of the intermicellar exchange, one can understand that the film flexibility parameter *f* and the *k_ex_* parameters for exchange of reactants (*k_ex,A_*, *k_ex,B_* and *k_ex,R_*) and non-aggregated products (*k_ex,P_*) have to be related. One expects that a flexible film not only implies a larger particle transfer (high *f*) but also a faster exchange of reactants and free products (high values of the *k_ex_* parameters). The simulation distinguishes the kind of interchanged species: The *k_ex_* simulation parameters govern the exchange of reactants and non-aggregated products, and they will be related to the dimer stability. On the other hand, the *f* parameter quantifies the maximum size of exchanged particle, so it is related to the interdroplet channel size. Both factors rise together, because an increase of the surfactant flexibility allows the exchange of larger particles and also a quicker exchange of free units. Therefore, in this study, a channel size *f* = 5, 30 and 90 is associated with *k_ex_* = 1, 5 and 15 free atoms/molecules which could be interchanged during a collision, respectively.

## 4. Results and Discussion

### 4.1. Simulation Results: Simple Particles

**Figure 1:**
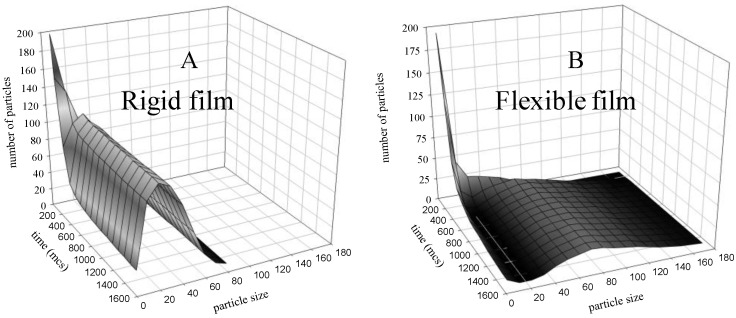
Time evolution of the number of droplets containing particles using different flexibility. Synthesis conditions: concentration 〈*c_A_*〉 = 〈*c_B_*〉 = 32 reactants in a droplet; chemical reaction rate v = 1, critical nucleus size n* = 1, no restriction by droplet size.(**A**) Rigid film, *f* = 5, *k_ex_* = 1. (**B**) Flexible film, *f* = 30, *k_ex_* = 5.

Figure 1 shows how the nanoparticle size distributions vary with time, simulating two different surfactants. The existence of two well-defined processes can be observed: the rapid formation of a large number of small nuclei, and the subsequent slower growth. By using different flexibilities, *i.e.*, by changing the interdroplet channel size and the intermicellar exchange rate, the final particle size can be modified, obtaining a broader distribution and a bigger mean size when the flexibility of the film is greater. Moreover, the polydispersity of particle sizes becomes bigger as the flexibility increases. Similar qualitative results have been obtained by using different combinations of the synthesis variables (concentration, droplet size, chemical rate, critical nucleus number).

**Figure 2:**
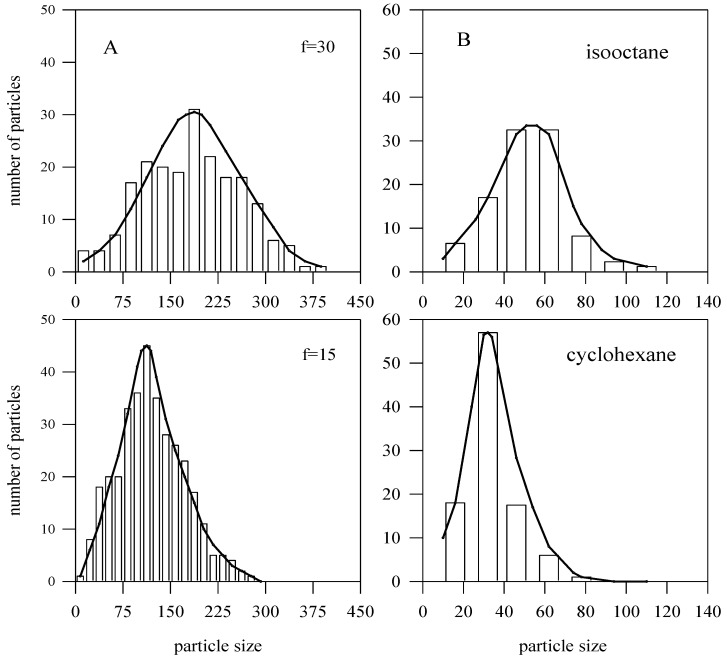
(**A**) Simulation results for 〈*c*〉 = 100 and different *f* values (*f* = 30, top panel; *f* = 15, bottom panel). (**B**) Size distributions of silver particles synthesized in isooctane (top) and cyclohexane (bottom) (data obtained from reference [55]). The lines are simply a guide for the eye.

These results are in keeping with the experimental results of Petit *et al.* [55], who studied the synthesis of silver nanoclusters, using different alkanes (isooctane and cyclohexane). As a matter of fact, the use of cyclohexane compared to isooctane as bulk solvent induces a decrease in the interaction potential, which decreases the intermicellar exchange rate constant by a factor of ten [56]. By using cyclohexane as bulk solvent, electron micrographs reveal a decrease in the size, polydispersity, and number of silver particles compared to that obtained with isooctane (see Figure 2). It should be noticed that no change in micellar size has been observed by SAXS. Hence, at a given water content, the decrease of the intermicellar exchange rate constant induces a decrease in particle size. The number of silver metallic particles obtained is lower than that observed by using isooctane as bulk solvent, indicating a diminution in the reduction yield. This is confirmed by a decrease in the 250 nm absorbance when using cyclohexane instead of isooctane. We can explain these results on the basis of our simulations. The interdroplet rate exchange in cyclohexane is lower than in isooctane [57]. The natural droplet curvature in cyclohexane is similar to the actual droplet curvature, because of the hard-sphere interactions. On the contrary, the natural droplet curvature in isooctane is smaller than the actual droplet curvature, because of the attractive interactions. This means that the film flexibility in cyclohexane is smaller than in isooctane, as can be observed in Figure 2, which shows the agreement between experimental and simulation results. The fact that larger particles are favored by ripening at high *f* values implies that the exchange rate will be quicker as *f* increases, and therefore, the effective rate constant for droplet communication will be greater [58]. One can conclude that an increase in the interdroplet channel size leads to bigger and polydisperse nanoparticle sizes.

**Figure 3:**
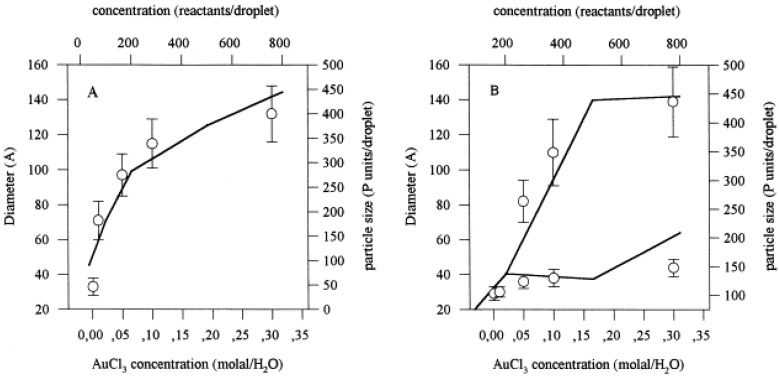
Particle size *versus* reactant concentration: (**A**) Symbols show Au particles prepared in DOBANOL-hexane-water; line shows simulation results (*f* = 30, droplet size = 500). (**B**) Symbols show Au particles prepared in PEDGE-hexane-water (data obtained from reference [59]); line shows simulation results (*f* = 5, droplet size = 500).

Another point of interest is the influence of surfactant on the kind of size distribution. Figure 3 shows the variation of the particle sizes as the concentration increases, using two different surfactants, *i.e.*, two different values of the film flexibility. Symbols in Figure 3A show the variation of Au particle diameter as a function of precursor AuCl_3_ concentration synthesized in DOBANOL-hexane-water microemulsion (data obtained from reference [59]). One can observed that particles of one size are obtained (unimodal particle size distribution), and size increases with increasing concentration. DOBANOL is a mixture of pentaethyleneglycolundecyl (<1 wt %), dodecyl (<41 wt %), tridecyl (<58 wt %) and tetradecyl (<1 wt %). As DOBANOL is composed of several surfactants, the water core will be more flexible than that obtained using a simple surfactant. This case is represented in Figure 3B, which shows results for the same reaction, at the same range of concentrations, but using PEDGE as a surfactant. In PEDGE-hexane-water microemulsion, at low concentration only small particles are formed, while both small and large particles are formed at high concentrations (bimodal particle size distribution). Lines in both figures show simulation results with varying the film flexibility parameter. The model simulation allows us to explain the fact that the same reaction using different surfactants leads to different types of distribution. If the film is flexible, the droplets can exchange the nuclei in such a way that larger droplets grow at expense of smaller ones (Ostwald ripening). These disappear, giving rise to unimodal distributions for all values of concentration. Likewise, at low concentration, the distribution is always unimodal, independent of film flexibility (see reference [33] for details). Therefore, only the combination of a rigid film and a quite high concentration gives rise to bimodal distributions. High concentrations mean that growth by autocatalysis and growth by ripening take place separately [58]. However, only when the film is sufficiently rigid is autocatalysis the most important method of growth (see Figure 7A in ref. [58]). Therefore, one can conclude that a bimodal distribution is obtained only if autocatalysis is the predominant manner of growth and if both growth processes (autocatalysis and ripening) do not take place simultaneously.

### 4.2. Simulation Results: Bimetallic Particles

**Figure 4:**
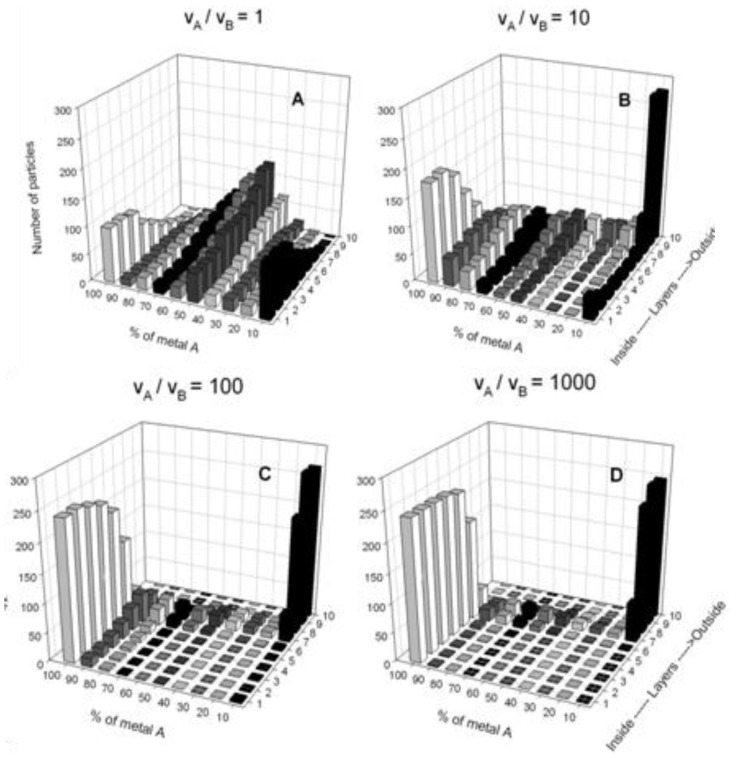
Number of particles *versus* the percentage of one of the products (*AM*), from the nanoparticle core to the outside (layer by layer) for different reduction rate ratios, and keeping the synthesis variables constant: reactant concentration 〈*c_A_*〉 = 〈*c_B_*〉 = 32, *f* = 5, *k_ex_* = 1, n* = 1, no restriction by droplet size. (**A**) v_A_/v_B_ = 1; (**B**) v_A_/v_B_ = 10; (**C**) v_A_/v_B_ = 100; (**D**) v_A_/v_B_ = 1000.

To illustrate the nanoparticle structure dependence on the difference in chemical reactions rates of both metals, we have performed computer simulations using different reduction rate ratios v_A_/v_B_, where v_A_ and v_B_ are the *A* metal and *B* metal reduction rates, respectively, and keeping the synthesis variables constant (reactants concentration, surfactant flexibility, droplet size). Figure 4 shows simulation results. The number of particles containing different percentage of one of the metals (*A*) is monitored from the nanoparticle core to the outside (layer by layer). As expected, when both reduction rates are equal (see Figure 4. A, v_A_/v_B_ = 1), an alloy structure of nanoparticle is observed from the core/beginning. Although the inner layers of some particles are mainly composed of only one of the metals, the composition shows a progressive improvement towards a perfect mix of both metals from the inner to the outer layers. At the end of the process, the composition of most of the particles is 50% in each metal. On increasing the difference between the chemical reaction rates, a progressive change from an alloy to a core-shell structure is observed (see Figure 4A to D). Figure 4D shows a core-shell structure (v_A_/v_B_ = 1000), in which the inner layers are composed of the metal with the fastest reduction rate, in the middle a few particles have a mixture, and the outer layers contain the metal with the slowest reduction rate. Similar qualitative results are obtained using different synthesis conditions.

**Figure 5:**
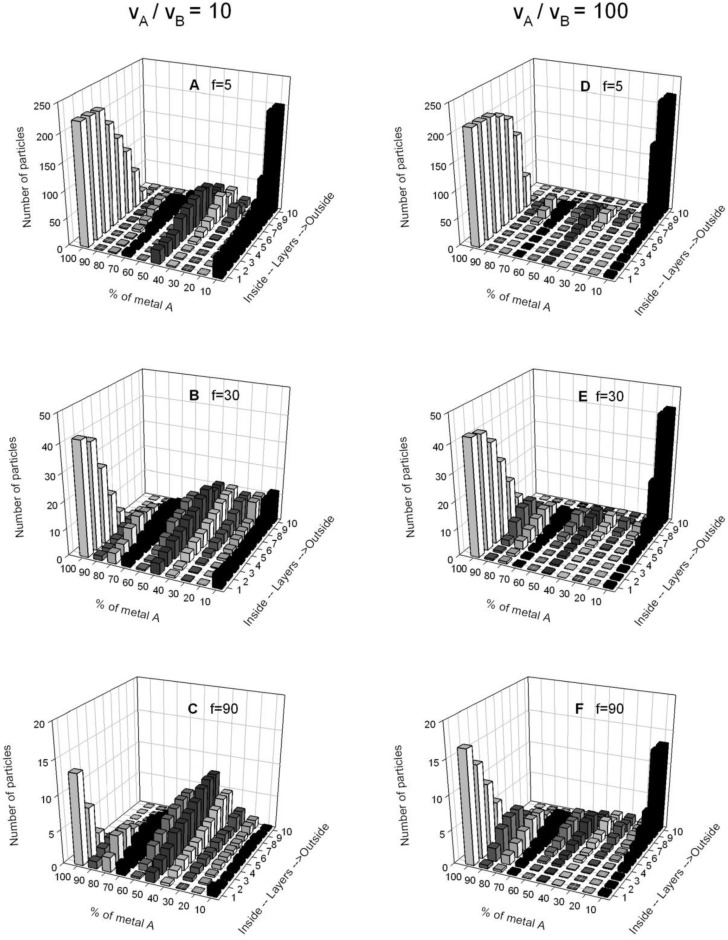
Number of particles *versus* the percentage of one of the products (*AM*), from the nanoparticle core to the outside (layer by layer) for different film flexibilities, and keeping the reduction rate ratio constant. Left column shows results using v_A,r_ / v_B,r_ = 10 and right column results using v_A,_/v_B_ = 100. Synthesis conditions: reactant concentration 〈*c_A_*〉 = 〈*c_B_*〉 = 4, n* = 1, no restriction by droplet size. (**A**) v_A_/v_B_ = 10, rigid film *f* = 5, *k_ex_* = 1; (**B**) v_A_/v_B_ = 10, flexible film *f* = 30, *k_ex_* = 5; (**C**) v_A_/v_B_ = 10, very flexible film *f* = 90, *k_ex_* = 15; (**D**) v_A_/v_B_ = 100, rigid film *f* = 5, *k_ex_* = 1; (**E**) v_A_/v_B_ = 100, flexible film *f* = 30, *k_ex_* = 5; (**F**) v_A_ /v_B_ = 100, very flexible film *f* = 90, *k_ex_* = 15.

A second point of interest is studying how the nanoparticle structure can be modified by changing the microemulsion composition. The effectiveness in material intermicellar exchange depends mainly on the surfactant film flexibility around the droplets, which governs the ease with which channels communicating colliding droplets can form, their stability and the size of these channels [25,54]. Previous simulation results of simple nanoparticles synthesized in microemulsions show that the effectiveness in material intermicellar exchange, determined by the microemulsion composition, plays a relevant role in the kinetics [60]. Likewise, the intermicellar exchange rate modifies the nanoparticle structure, as can be seen in Figure 5. It shows the bimetallic nanoparticle structures obtained by simulation using different film flexibility parameters, keeping concentration and chemical reaction rates constant. When a rigid film is used (Figure 5A and D), a core-shell structure is obtained, with the metal separation being more pronounced as the difference in reduction rate is larger, as was expected. As the film flexibility increases (see from up to down), the degree of alloying increases too. Note that a transition from core-shell to alloy structure is obtained for moderate differences in reduction rate (v_A_/v_B_ = 10, Figure 5 A, B and C). However, if both reductions take place at similar rates or if the difference between rates is large enough (v_A_/v_B_ ≥ 100), the structure is not modified by the surfactant film flexibility.

In order to compare simulation results with experimental ones, Table 1 shows experimental data of Au-Ag [61,62] and Au-Pt [63,64] nanoparticles. It is observed that a given bimetallic nanoparticle can be obtained in alloy form if the surfactant film flexibility is high, or in a core-shell structure using a rigid film. Good agreement between both kinds of results allows us to propose that it is possible, even when the chemical reaction rates are different, to obtain nanoalloyed nanoparticles just by changing the microemulsion composition. Once more, the dynamic character of the microemulsion droplets is the key factor to explain this behavior (see reference [65] for details).

**Table 1:** Bimetallic nanoparticles prepared by simultaneous reduction in microemulsions.

Metals	Structure	Microemulsion reductor agent; metal precursor	*f*	Ref
Au-Ag	Au core-enriched in Ag shell	water/AOT/isooctane N_2_H_5_OH; Ag^+^, AuCl_4_^-^	rigid	[62]
	nanoalloy	water/TritonX-100/cyclohexane NaBH_4_, Ag^+^, AuCl_4_^-^	flexible	[61]
Au-Pt	core-shell	water/AOT/isooctane N_2_H_5_OH, AuCl_4_^-^, PtCl_6_^2-^	rigid	[63]
	nanoalloy	water/Tergitol 15-S-5/isooctane N_2_H_5_OH, AuCl_4_^-^, PtCl_6_^2-^	flexible	[64]

## 5. Conclusions

The above results suggest that microemulsion composition strongly affects size, polydispersity, type of size distribution and structure of nanoparticles synthesized in microemulsions. One can conclude that an increase in surfactant flexibility leads to bigger and polydispersed nanoparticle sizes. In addition, the same reaction using different surfactants leads to different types of distribution, with a bimodal distribution obtained at high concentrations and by using rigid films.

In relation to bimetallic nanoparticles, if the nanoparticle is composed of two metals with a moderate difference in reduction potentials, increasing the surfactant flexibility modifies the nanoparticle structure, giving rise to a transition from a nanoalloy (using a rigid film) to a core-shell structure (using a flexible one).

Good agreement between experimental and simulations results show the validity of the simulation model used in this study.

## References

[1] López-Quintela M.A. (2003). Synthesis of nanomaterials in microemulsions: Formation mechanisms and growth control. Curr. Opin. Colloid Interface Sci..

[2] Nagabhushana K.S., Bönnemann H., Zhou B., Hermans S., Somorjai G.A. (2004). Wet chemical synthesis of nanoparticles. Nanotechnology in Catalysis.

[3] Sun S., Murray C.B., Weller D., Folks L., Moser A. (2000). Monodisperse FePt nanoparticles and ferromagnetic FePt nanocrystal superlattices. Science.

[4] Toshima N., Yonezawa T., Kushihashi K. (1993). Polymer-protected palladium-platinum bimetallic clusters: Preparation, catalytic properties and structural considerations. J. Chem. Soc. Faraday Trans..

[5] Chung Y.M., Rhee H.K. (2004). Dendrimer-templated Ag-Pd bimetallic nanoparticles. J. Colloid Interface Sci..

[6] Han S.W., Kim Y., Kim K. (1998). Dodecanethiol-derivatized Au/Ag bimetallic nanoparticles: TEM, UV/VIS, XPS, and FTIR analysis. J. Colloid Interface Sci..

[7] Capek I. (2004). Preparation of metal nanoparticles in water-in-oil (w/o) microemulsions. Adv. Colloid Interface Sci..

[8] Yadav O.P., Palmqvist A., Cruise N., Holmberg K. (2003). Synthesis of platinum nanoparticles in microemulsions and their catalytic activity for the oxidation of carbon monoxide. Colloids Surf. A.

[9] Sánchez-Dominguez M., Boutonnet M., Solans C. (2009). A novel approach to metal and metal oxide nanoparticle synthesis: The oil-in-water microemulsion reaction method. J. Nanopart. Res..

[10] Stubenrauch C., Wielpütz T., Sottmann T., Roychowdhury C., DiSalvo F.J. (2008). Microemulsions as templates for the synthesis of metallic nanoparticles. Colloids Surf. A.

[11] Arriagada F.J., Osseo-Asare K. (1999). Synthesis of Nanosize Silica in a Nonionic Water-in-Oil Microemulsion: Effects of the Water/Surfactant Molar Ratio and Ammonia Concentration. J. Colloid Interface Sci..

[12] Bumajdad A., Eastoe J., Zaki M.I., Heenan R.K., Pasupulety L. (2007). Generation of metal oxide nanoparticles in optimised microemulsions. J. Colloid Interface Sci..

[13] Hammouda A., Gulik T., Pileni M.P. (1995). Synthesis of Nanosize Latexes by Reverse Micelle Polymerization. Langmuir.

[14] Summers M., Eastoe J., Davis S. (2002). Formation of BaSO4 Nanoparticles in Microemulsions with Polymerized Surfactant Shells. Langmuir.

[15] Ethayaraja M., Bandyopadhyaya R. (2006). Population Balance Models and Monte Carlo Simulation for Nanoparticle Formation in Water-in-Oil Microemulsions: Implications for CdS Synthesis. J. Am. Chem. Soc..

[16] Pillai V., Kumar P., Hou M.J., Ayyub P., Shah D.O. (1995). Preparation of nanoparticles of silver halides, superconductors and magnetic materials using water-in-oil microemulsions as nano-reactors. Adv. Colloid Interface Sci..

[17] Viger M., Live L., Therrien O., Boudreau D. (2008). Reduction of self-quenching in fluorescent silica-coated silver nanoparticles. Plasmonics.

[18] Jain R., Shukla D., Mehra A. (2006). A Monte Carlo Model for the Formation of Core-Shell Nanocrystals in Reverse Micellar Systems. Ind. Eng. Chem. Res..

[19] Shukla D., Mehra A. (2006). Modeling Shell Formation in Core-Shell Nanocrystals in Reverse Micelle Systems. Langmuir.

[20] Wanjala B.N., Luo J., Loukrakpam R., Fang B., Mott D., Njoki P.N., Engelhard M., Naslund H.R., Wu J.K., Wang L., Malis O., Zhong C.J. (2010). Nanoscale alloying, phase-segregation, and core-shell evolution of Gold-Platinum nanoparticles and their electrocatalytic effect on oxygen reduction reation. Chem. Mater..

[21] Holmberg K. (2004). Surfactant-templated nanomaterials synthesis. J. Colloid Interface Sci..

[22] Lisiecki I., Pileni M.P. (1993). Synthesis of copper metallic clusters using reverse micelles as microreactors. J. Am. Chem. Soc..

[23] Shah P.S., Holmes J.D., Johnston K.P., Korgel B.A. (2002). Size-Selective Dispersion of Dodecanethiol-Coated Nanocrystals in Liquid and Supercritical Ethane by Density Tuning. J. Phys. Chem. B.

[24] Kitchens C.L., McLeod M.C., Roberts C.B. (2003). Solvent Effects on the Growth and Steric Stabilization of Copper Metallic Nanoparticles in AOT Reverse Micelle Systems. J. Phys. Chem. B.

[25] Quintillán S., Tojo C., Blanco M.C., López-Quintela M.A. (2001). Effects of the Intermicellar Exchange on the Size Control of Nanoparticles Synthesized in Microemulsions. Langmuir.

[26] Tojo C., Barroso F., de Dios M. (2006). Critical nucleus size effects on nanoparticle formation in microemulsions: A comparison study between experimental and simulation results. J. Colloid Interface Sci..

[27] de Dios M. (2009). Síntesis de nanopartículas en microemulsiones. Estudio por simulación. Ph.D. Thesis.

[28] Curl R.L. (1963). Dipersed phase mixing. Theory and effects in simple reactors. AIChE J..

[29] Niemann N., Rauscher F., Adityawarman D., Voigt A., Sundmacher K. (2006). Microemulsion-assisted precipitation of particles: Experimental and model-based process analysis. Chem. Eng. Process.

[30] Niemann B., Veit P., Sundmacher K. (2008). Nanoparticle precipitation in reverse microemulsions: Particle formation dynamics and tailoring of particle size distributions. Langmuir.

[31] Sundmacher K., Niemann B. (2010). Nanoparticle precipitation in microemulsions: Population balance model and identification of bivariate exchange kernel. J. Colloid Interface Sci..

[32] La Mer V.K., Dinegar R.H. (1950). Theory, production, and mechanism of formation of monodispersed hydrosols. J. Am. Chem. Soc..

[33] Tojo C., Blanco M.C., López-Quintela M.A. (1997). Preparation of Nanoparticles in Microemulsions: A Monte Carlo Study of the Influence of the Synthesis Variables. Langmuir.

[34] Hellweg T. (2002). Phase structures of microemulsions. Curr. Opin.Colloid Interface Sci..

[35] Bagwe R.P., Khilar K.C. (1997). Effects of the Intermicellar Exchange Rate and Cations on the Size of Silver Chloride Nanoparticles formed in Reverse Micelles of AOT. Langmuir.

[36] Bagwe R.P., Khilar K.C. (2000). Effects of Intermicellar Exchange Rate on the Formation of Silver Nanoparticles in Reverse Microemulsions of AOT. Langmuir.

[37] Cason J., Miller M.E., Thompson J.B., Roberts C.B. (2001). Solvent effects on copper nanoparticle growth behavior in AOT reverse micelle systems. J. Phys. Chem. B.

[38] De Smet Y., Deriemaecker L., Finsy R. (1997). A Simple Computer Simulation of Aging Processes. Langmuir.

[39] Taisne L., Cabane B. (1998). Emulsification and Ripening following a Temperature Quench. Langmuir.

[40] Zhang W., Qiao X., Chen J. (2007). Synthesis of nanosilver colloidal particles in water/oil microemulsion. Colloids Surf. A.

[41] Ethayaraja M., Ravikumar C., Muthukumaran D., Dutta K., Bandyopadhyaya R. (2007). CdS-ZnS Core-Shell Nanoparticle Formation: Experiment, Mechanism, and Simulation. J. Phys. Chem. C.

[42] Henle J., Simon P., Frenzel A., Scholz S., Kaskel S. (2007). Nanosized BiOX (X = Cl, Br, I) Particles Synthesized in Reverse Microemulsions. Chem. Mater..

[43] Kimijima K., Sugimoto T. (2005). Effects of the water content on the growth rate of AgCl nanoparticles in a reversed micelle system. J. Colloid Interface Sci..

[44] Monnoyer Ph., Fonseca A., Nagy J.B. (1995). Preparation of colloidal AgBr particles from microemulsions. Colloids Surf. A.

[45] Koutzarova T., Kolev S., Ghelev Ch., Paneva D., Nedkov I. (2006). Microstructural study and size control of iron oxide nanoparticles produced by microemulsion technique. Phys. Status Solidi C.

[46] Destrée C., Ghijsen J., Nagy J.B. (2007). Preparation of Organic Nanoparticles Using Microemulsions: Their Potential Use in Transdermal Delivery. Langmuir.

[47] Tojo C., Blanco M.C., López-Quintela M.A. (1998). Microemulsions as microreactors: A Monte Carlo simulation on the synthesis of particles. J. Non-Cryst. Solids.

[48] López-Quintela M.A., Tojo C., Blanco M.C., García Río L., Leis J.R. (2004). Microemulsion dynamics and reactions in microemulsions. Curr. Opin. Colloid Interface Sci..

[49] Curri M.L., Agostiano A., Manna L., Della Monica M., Catalano M., Chiavarone L., Spagnolo V., Lugarà M. (2000). Synthesis and Characterization of CdS Nanoclusters in a Quaternary Microemulsion: The Role of the Cosurfactant. J. Phys. Chem. B.

[50] Andelman D., Cates M.E., Roux D., Safran S.A. (1987). Structure and phase equilibrium of microemulsions. J. Chem. Phys..

[51] Cates M.E., Andelman D., Safran S.A., Roux D. (1988). Theory of microemulsions: Comparison with experimental behaviour. Langmuir.

[52] Szleifer I., Kramer D., Ben-Shaul A., Roux D., Gelbart W.M. (1988). Curvature elasticity of pure and mixed surfactant films. Phys. Rev. Lett..

[53] Jain T.K., Cassin G., Badiali J.P., Pileni M.P. (1996). Relation between Exchange Process and Structure of AOT Reverse Micellar System. Langmuir.

[54] López-Quintela M.A., Rivas J., Blanco M.C., Tojo C., Liz Marzán L., Kamat P.V. (2003). Synthesis of nanoparticles in microemulsions. Nanoscale Materials.

[55] Petit C., Lixon P., Pileni M.P. (1993). In situ synthesis of silver nanocluster in AOT reverse micelles. J. Phys. Chem. B.

[56] Towey T.F., Khan-Lodhi A., Robinson B.H. (1990). Kinetics and mechanism of formation of quantum-sized cadmium sulfide particles in water-Aerosol OT-oil microemulsions. J. Chem. Soc. Faraday Trans..

[57] Fletcher D.I., Howe A.M., Robinson B.H. (1987). The kinetics of solubilisate exchange between water droplets of a water-in-oil microemulsion. J. Chem. Soc. Faraday Trans..

[58] Tojo C., Rivadulla F., Blanco M.C., López-Quintela M.A. (1997). Kinetics of the Formation of Particles in Microemulsions. Langmuir.

[59] Nagy J.B., Barette D., Fonseca A., Jenieau L., Monnoyer P.H., Piedigrosso P., Ravet-Bodart I., Verfaillie J.-P., Wathelet A., Fendler J.H., Dékány I. (1996). Nanoparticles in Microemulsions: A General Approach. Nanoparticles in Solids and Solutions.

[60] de Dios M., Barroso F., Tojo C., Lopez-Quintela M.A. (2009). Simulation of the kinetics of nanoparticle formation in microemulsions. J. Colloid Interface Sci..

[61] Pal A., Shah S., Devi S. (2007). Preparation of silver, gold and silver-gold bimetallic nanoparticles in w/o microemulsion containing TritonX-100. Colloids Surf. A.

[62] Chen D., Chen C. (2002). Formation and characterization of Au-Ag bimetallic nanoparticles in water-in-oil microemulsions. J. Mater. Chem..

[63] Hernández-Fernández P., Rojas S., Ocón P., Gómez de la Fuente J.L., San Fabián J., Sanza J., Peña M.A., García-García F.J., Terreros P., Fierro J.L.G. (2007). Influence of the Preparation Route of Bimetallic Pt-Au Nanoparticle Electrocatalysts for the Oxygen Reduction Reaction. J. Phys. Chem. C.

[64] Wu M., Chen D., Huang T. (2001). Preparation of Au/Pt bimetallic nanoparticles in water-in-oil microemulsions. Chem. Mater..

[65] Tojo C., de Dios M., López-Quintela M.A. (2009). On the structure of bimetallic nanoparticles synthesized in microemulsions. J. Phys. Chem. C.

